# A prospective cohort study to investigate the transmission and burden of Staphylococcus aureus in Sri Lanka

**DOI:** 10.1099/mgen.0.001336

**Published:** 2024-12-19

**Authors:** T. Locke, S. Siribaddana, J.A.A.S. Jayaweera, C.M. Suligoy, T.I. de Silva, R.M. Corrigan, T.C. Darton

**Affiliations:** 1 Division of Clinical Medicine, School of Medicine and Population Health, The University of Sheffield, Sheffield, UK; 2 The Florey Institute of Infection, The University of Sheffield, Sheffield, UK; 3 Department of Medicine, Faculty of Medicine and Allied Sciences, Rajarata University of Sri Lanka, Saliyapura, Sri Lanka; 4 Department of Microbiology, Faculty of Medicine and Allied Sciences, Rajarata University of Sri Lanka, Saliyapura, Sri Lanka; 5 School of Biosciences, The University of Sheffield, Sheffield, UK

**Keywords:** Asia, colonization, community-acquired infection, healthcare-acquired infection, *Staphylococcus aureus*, transmission

## Abstract

Methicillin-resistant *Staphylococcus aureus* (MRSA) is a common cause of infection in both community and healthcare settings, and the household may be a central component linking these two environments. Strategies to prevent *S. aureus* transmission and thereby reduce the risk of infection must be informed by a detailed understanding of local epidemiology. These data are typically lacking in many low- and middle-income countries. Therefore, we aimed to investigate the circulation of infecting *S. aureus* strains in Sri Lanka, with a focus on the community and healthcare interface. A prospective longitudinal cohort study was performed between July and December 2021. Index patients with *S. aureus* infection and up to four of their household contacts were enrolled in the study. Colonization was assessed by sampling participants’ nose and axilla at two time points over 3 months of follow-up. Whole-genome sequencing (WGS) was used to characterize isolates and assess strain similarity to identify transmission episodes and environmental clusters. A total of 153 participants were recruited, including 42 *S*. *aureus*-positive index patients and 111 household contacts. The baseline prevalence of *S. aureus* colonization amongst household contacts was 11.7% (13/111), of which 30.8% (4/13) were methicillin-resistant. A total of 88 *S*. *aureus* isolates underwent WGS and three multilocus sequence types predominated: ST672, ST5 and ST6. Each type had unique virulence characteristics but was identified in both community and healthcare environments. Colonization of household members with the index’s infecting strain was not detected. *S. aureus* is a major cause of morbidity and mortality in low-resource settings such as Sri Lanka, yet little is known about risk factors and transmission networks. In this descriptive study, we have identified a small number of strains that appear to be well established and capable of causing both severe infection and asymptomatic colonization. Transmission of *S. aureus* did not appear to be occurring frequently in the household, and, therefore, preventative strategies that target high-risk groups may be more successful than universal community-based measures.

## Data Summary

The authors confirm all supporting data and protocols have been provided within the article or through supplementary data files. Genome assemblies have been uploaded to BioProject PRJNA1093472, under BioSample numbers SAMN40655892 to SAMN40655984 (see Table S1).

**Impact Statement**

*Staphylococcus aureus* infections pose a significant health burden in many low- and middle-income countries (LMIC). Mortality rates can be high, and treatment is frequently complicated by high rates of antimicrobial resistance. Virulent strains are now found in both healthcare and community settings. Data from high-income settings suggest that the household is an important site of transmission and that interventions targeted here can prevent further infections. However, there is a relative lack of comparable data from LMIC. Here, we address these gaps by performing a longitudinal cohort analysis involving 153 participants from North Central Sri Lanka, an area with a high burden of methicillin-resistant *S. aureus*. This study provides valuable insights into the epidemiology of *S. aureus* in a low- and middle-income setting, with a specific focus on the transmission dynamics between community and healthcare settings. We identified three dominant strains, each with unique virulence characteristics and present in both environments. Interestingly, household transmission of infecting strains was not frequently detected, suggesting that the value of expensive household decolonization strategies in resource-limited settings may be limited. We believe that the findings in this study will contribute to the growing discourse around *S. aureus* transmission networks and infection prevention.

## Introduction

*Staphylococcus aureus* is a critically important human pathogen capable of causing both asymptomatic colonization or invasive infection. *S. aureus* is the leading cause of death due to bacterial infection worldwide, frequently due to antibiotic-resistant strains such as methicillin-resistant *S. aureus* (MRSA) [1,2]. Whilst MRSA infection was initially linked to healthcare exposure, MRSA is now equally recognized as a community-associated pathogen in most regions [3]. This shift in epidemiology was initially driven by distinct clones; however, the distinction between community-associated (CA) and healthcare-associated (HA) strains is now less clear-cut [3]. For example, the emergence of the USA300 strain in the USA was linked to increasing rates of CA-MRSA skin and soft tissue infection (SSTI) but later also spread into healthcare settings [4]. The exact mechanism behind this mixing of transmission environments remains unclear, although it seems likely that colonization, infection and population movement serve to link the two environments.

In high-income settings, the household plays a central role in perpetuating *S. aureus* transmission networks [5]. Therefore, in high-transmission settings, targeted household interventions could play a role in reducing the global burden of *S. aureus* infection. In the absence of other effective interventions to prevent colonization, such as vaccination, options to interrupt networks are currently limited to oral or topical antimicrobials or environmental disinfectants. Topical decolonization therapy reduces the risk of secondary cases of infection within the household but is typically expensive and not readily available in all regions [6]. Community-level intervention is likely to offer the most benefit in areas with high rates of invasive infection and antimicrobial resistance (AMR). However, identifying these communities is particularly challenging in many low- and middle-income countries (LMIC) due to the limited availability of *S. aureus* surveillance data. Where *S. aureus* surveillance data is available, LMIC in Asia has some of the highest proportion of AMR strains in the world [7,8]. This is particularly true of Sri Lanka, where MRSA has previously been shown to cause nearly 90% of HA *S. aureus* infections [7]. A dominant sequence type (ST) 5, MRSA, Panton–Valentine leukocidin (PVL) positive clone has previously been identified in hospitalized patients diagnosed with both CA and HA infections [9]. Phylogenetic analysis pointed to the more widespread circulation of this strain outside of Sri Lanka, which led us to hypothesize that community colonization and transmission may be acting as a reservoir that drives and sustains spread into the hospital environment.

In this study, we aimed to investigate colonization and transmission of infecting *S. aureus* strains in North Central Sri Lanka, with a particular focus on the community and healthcare interface. Secondly, we describe the molecular and phenotypic epidemiology of infecting and colonizing strains in this dynamic, high-incidence setting.

## Methods

### Study design

A prospective cohort study was performed at Anuradhapura Teaching Hospital, in the North Central Province of Sri Lanka, between July and December 2021. The hospital serves a large geographical catchment area, with an approximate population size of 1.5 million. Potential index participants (IP) were identified through a review of hospital microbiology records for a current episode of culture-confirmed *S. aureus* infection.

Inclusion criteria for IP included (1) adults over 18 years old, (2) with culture-confirmed *S. aureus* infection diagnosed at the Anuradhapura General Hospital, (3) who lived within 1-day travel of the hospital and (4) have provided informed consent for participation in the study. Due to the longitudinal nature of the study, potential IP were excluded if deemed unsuitable by the responsible clinician, typically due to their co-morbidities and overall performance status.

After the recruitment of the IP to the study, available household contacts (HC) were then approached for inclusion in the study. HC were (1) resident in the same household as the IP, (2) willing and able to give informed consent, (3) likely to remain resident in the same household for at least 3 months from the date of recruitment and (4) able to comply with study requirements as judged by the research team. The IP’s initial infection was categorized as either community-acquired (onset in the community or within 48 h of hospital admission) or hospital-acquired (onset more than 48 h after admission).

For the purposes of sample size calculations, we anticipated a prevalence of *S. aureus* colonization between 20 and 50%. Using Cochran’s formula for sample size estimation, we calculated that a sample size of 250 would yield an acceptable margin of error, ranging from 0.0496 to 0.0620, at a 95% confidence interval. Following a review of the local laboratory and average household size data, we aimed to recruit 50 IP and up to 4 HC per IP, providing a maximum sample size of 250.

### Data collection/sampling

There were two assessment timepoints: baseline (V1) occurred as soon after enrolment as possible and follow-up (V2) at 3 (±1) months. At both timepoints, study staff collected demographic, clinical and social/lifestyle data using a standardized collection form. *S. aureus* colonization was assessed by sampling the anterior nares and axilla with in-house produced sterile cotton-tipped swabs. These were produced by trained laboratory technicians and sterilized by autoclaving at 121 °C for 30 min with autoclave indicator strips used for quality assurance. This occurred at both timepoints for HC and at V2 only for IP. Anterior nares samples were collected by introducing a swab 2–3 cm into both nostrils and rotating four times. Using a separate swab, the axilla skin was sampled unilaterally. Swabs were transported to the Rajarata University of Sri Lanka, stored at 4 °C and processed within 24 h. The visits for each household were conducted on a single day and were, therefore, scheduled to maximize participant availability.

### Sample processing and *S. aureus* identification

Colonization swabs had an initial enrichment step with overnight culture in brain heart infusion broth supplemented with 7.5% NaCl. Aerobic blood culture bottles were incubated and monitored at 37 °C for 48 h using the fully automated BD BACTEC FX system (Becton Dickinson, USA). Positive blood culture samples, along with all other clinical specimens and the colonization swabs, were then processed in the same manner. Firstly, they were sub-cultured to blood, chocolate and MacConkey agar plates (HiMedia, India) with overnight incubation at 37 °C. Isolates were provisionally identified as *S. aureus* if they met the following criteria: (1) Gram-positive cocci observed by microscopy, (2) catalase positive (bubbling observed on mixing of the isolate with 3% hydrogen peroxide), (3) coagulase positive (coagulation observed on mixing of the isolate with plasma) and (4) DNAse test positive (clear zone around colonies when grown on DNAse test agar [HiMedia, India]). These isolates were then sub-cultured on mannitol salt agar (MSA) and confirmed as *S. aureus* if yellow colonies were visible after overnight incubation. Three to five representative colonies were then frozen at −20 °C and later shipped at 4 °C in stab agar tubes to the University of Sheffield, UK. Upon receipt, they were sub-cultured on MSA to confirm purity, and a confirmatory coagulase test was performed (Pastorex Staph Plus Latex Agglutination, Bio-Rad, USA). Three to five representative colonies were frozen at −80 °C (Microbank, Pro-Lab Diagnostics, Canada), and all further work was conducted on sub-cultures of this frozen stock.

### Antibiotic sensitivity testing

Antibiotic sensitivity testing was performed on clinical isolates using the Kirby–Bauer method of disc diffusion according to Clinical and Laboratory Standards Institute standards [10]. Vancomycin and mupirocin susceptibility testing was performed on all isolates using gradient diffusion strips (Biomerieux ETEST). The minimum inhibitory concentration (MIC) values were interpreted with EUCAST breakpoints, with resistance defined as an MIC >2 mg l^−1^ for vancomycin and ≥8 mg l^−1^ for mupirocin [11,12].

### Biofilm formation

Biofilm formation was quantified using a modified version of previously described assays [13,14]. In brief, an overnight culture of bacteria was diluted in tryptic soy broth (TSB) with 1% glucose to an OD_600_ of 0.05. This was incubated stationary at 37 °C overnight in a microplate (Nunc MicroWell, Thermo Fisher Scientific). Non-inoculated TSB/1% glucose was used as a negative control, and the *S. aureus* RN4220 strain was a positive control. The absorbance of light at 595 nm was read in a multiplate reader (Varioskan LUX, Thermo Fisher Scientific) at two timepoints: (1) immediately after 24-h incubation (final culture density) and (2) after biofilm had been stained with 0.1% crystal violet and then dissolved with 33% glacial acetic acid. Each isolate was tested in triplicate on 2 consecutive days. Outliers were detected using the *Z*-score method. Readings were adjusted by subtracting the negative control value and expressed relative to their individual final culture density. Biofilm capability was categorized relative to the positive control and included strong (≥75%), moderate (25–74%) and weak (<25%).

### Whole-genome sequencing

Whole-genome sequencing (WGS) of bacteria was performed by MicrobesNG (Birmingham, UK). All isolates provisionally identified as *S. aureus* by culture/phenotypic methods were sequenced on the Illumina NovaSeq 6000 platform with 2×250 bp paired-end reads (Illumina, San Diego, CA). The sequences were trimmed (Trimmomatic with adapter removal and a sliding window quality cutoff of Q15), then *de novo* assembled (SPAdes) and annotated (Prokka) [15,17]. Quality control was performed using FastQC and Kraken (only including isolates with ≥90% reads classified as *S. aureus*) [18,19]. Genes associated with antimicrobial resistance and virulence factors were identified using the ABRicate tool with the Bacterial Antimicrobial Resistance Reference Gene Database and Virulence Factor Database, respectively [20,22]. Typing methods included SCC*mec* (staphopia-sccmec), spa (spaTyper) and multilocus sequence typing (MLST) (software using the *S. aureus* PubMLST database) [23,26]. The pangenome was determined using Roary with core genes defined as those present in ≥99% or more of the genomes [27]. Strain relatedness was determined by identifying SNP in the core genome alignment using SNP-sites, and pairwise genetic distances were calculated using SNP-dists [27,28]. Strains with a cgSNP distance of 15 or less were considered to be identical [29]. Maximum likelihood phylogenetic trees were constructed with FastTree using a generalized time-reversible model without bootstrapping based on 51 952 SNPs from 1672 core genes [30]. The phylogenetic tree was then visualized and annotated using the ggtree package in R [31].

### Statistical methods

Univariate risk factor analysis is presented as an odds ratio with 95% confidence intervals. *P*-values were calculated using Pearson’s chi-squared test, Fisher’s exact test or Mann–Whitney *U* test, and a value of less than 0.05 was deemed statistically significant. False discovery rate correction for multiple testing was applied. All data processing and analysis were performed using R version 4.3.1 [32].

## Results

### Recruitment summary

Seventy-seven IP were recruited between May and December 2021. Thirty-five (45.5%) of these were subsequently excluded from the assessment of *S. aureus* transmission, either because WGS did not confirm the infecting isolate to be *S. aureus* (8/35) or no associated HC were available for recruitment (27/35). Isolates from the latter group are referred to as additional participants (AP) and were included in the genomic and ST672 (where applicable) analysis, to give a broader context to the types of *S. aureus* strains causing invasive infection. The final study population was, therefore, 180 participants which comprised 42 IP, 111 HC and 27 AP. Of the 153 participants in the IP and HC groups, 79.1% (121/153) completed all study activities. This included 39/42 IP (2 died and 1 left the region after V1) and 82/111 HC (after V1, 5 withdrew from the study and 6 left the region; eighteen were unavailable at V2).

### Demographics and household characteristics

Amongst 42 households, a median of 3 HC (interquartile range [IQR] 1.3–3.8) per household was recruited, comprising 2 adults (IQR 1–3) and 1 child (IQR 1–2). HC were typically younger (median 33 vs. 47 years old, *P*<0.001) and more commonly female (64/111, 57.7% vs. 14/42, 33.3%; *P*=0.007) compared with IP (Table 1). The majority of HC were over 18 years old (81/111, 72.9%). Index participants had higher rates of hospitalization and medical procedures in the 12 months prior to study recruitment than HC. Medical co-morbidities were also more common, including diabetes mellitus and skin disorders.

**Table 1.** Baseline characteristics for index and household contact participants

Characteristics	Index, *N*=42*	Household contact, *N*=111*	*P*-value†	*q*-value‡
Demographics				
Female	14 (33%)	64 (58%)	**0.007**	0.014
Age – median (IQR)	47 (38, 58)	33 (16, 50)	**<0.001**	0.001
Medical event in the last 12 months				
Infection	7 (17%)	15 (14%)	0.6	0.7
Hospitalized	23 (55%)	3 (2.7%)	**<0.001**	<0.001
Outpatients	22 (52%)	64 (58%)	0.6	0.7
Medical procedure	24 (57%)	5 (4.5%)	**<0.001**	<0.001
Past medical history				
Alcoholism	4 (9.5%)	2 (1.8%)	**0.049**	0.073
Chronic kidney disease	4 (9.5%)	2 (1.8%)	**0.049**	0.073
Chronic lung disease	1 (2.4%)	2 (1.8%)	>0.9	>0.9
Connective tissue/inflammatory disease	2 (4.8%)	3 (2.7%)	0.6	0.7
Diabetes mellitus	15 (36%)	2 (1.8%)	**<0.001**	<0.001
Skin abnormality	7 (17%)	2 (1.8%)	**0.002**	0.004

* *n* (%); median (IQR).

† Pearson’s chi-squared test; Wilcoxon rank sum test; Fisher’s exact test.

‡ False discovery rate correction for multiple testing.

Statistically significant results (*P* < 0.05) are indicated in bold.

Houses were typically constructed with high-quality building materials, including brick walls (95.2%, 40/42), tiles or asbestos roofing (85.7%, 36/42) and synthetic or cement flooring (92.9%, 39/42). The majority of households had access to clean water, most commonly filtered (30/42, 71.4%) or piped (9/42, 21.4%). In contrast, toilet facilities were lower quality, with 65.9% (27/41, data missing for one house) using a pit latrine. Animal ownership was common, with over 90% of houses (39/42, 92.9%) owning at least one animal and 40.5% (17/42) with multiple. Domestic animals (typically cats and dogs) were more common than livestock ownership (most commonly cows and chickens) (38/42 vs. 7/42, 6/42 had both).

### Index participant infection characteristics

SSTI accounted for the majority of confirmed index infections (34/42, 81.0%). The limbs were the most common site of SSTI (20/34, 58.8%). Other infection syndromes included isolated bacteraemia (3/42, 7.1%), surgical site (3/42, 7.1%), gynaecological (1/42, 2.4%) and respiratory tract infections (1/42, 2.4%) (Table S2, available in the Supplementary Material). CA infection was more common than HA (34/42, 81.0% vs. 8/42, 19.0%). Nearly two-thirds (22/34, 64.7%) of those with CA infection had a history of healthcare contact within the last year, notably 44.1% (15/34) with an episode of hospital admission. HA *S. aureus* infection was diagnosed a median 9.5 (IQR 5.0–19.2) days after hospital admission. The *mecA* gene was identified in 22/42 (52.4%) of clinical isolates and 19/22 (86.4%) of these were resistant to cefoxitin. The median duration of hospital admission was 4.5 days (IQR 2.0–7.8, data not available for 20/42) and 4 required admission to the intensive care unit. Infection outcomes at V2 included 92.9% (39/42) cured with two deaths and one ongoing infection.

### *S. aureus* colonization

To assess for *S. aureus* colonization, a total of 463 swab samples were collected from study participants. These consisted of 221 samples of HC at V1 (one HC declined nasal sampling) and 242 samples taken at V2 (HC: 164/242 and IP: 78/242). The median interval between the index clinical sample and HC V1 sample was 25 days (IQR 12–49). Using culture and phenotypic methods, the baseline prevalence of *S. aureus* colonization amongst HC at V1 was 15.3% (17/111). However, 4/17 isolates were subsequently identified as *Staphylococcus argenteus* by WGS. Therefore, the final colonization prevalence of HC at V1 was 11.7% (13/111), with *mecA* present in 30.8% (4/13). This comprises 6/13 positive swabs collected from axilla, 7/13 positive from the nasal sample and none with both sites positive. The median interval between the V1 and V2 sampling was 79 days (IQR 70–92). At V2, the final HC colonization prevalence was 6.1% (5/82) (excluding one *S. argenteus*), and only one HC was positive at both timepoints. A single IP (1/39, 2.6%) was colonized with *S. aureus* at V2. Household contacts were sub-classified as colonized (positive at one or both timepoints) or non-colonized to assess colonization risk factors (Table 2). Univariate analysis identified female sex (odds ratio [OR] 3.04, 95% confidence interval [CI] 1.09–9.89, *P*=0.04) as weakly associated with HC colonization. At the household level, 19/42 (45.2%) houses were classified as positive as they had at least one colonized HC. The density of household positivity was generally low with 25–49% of HC colonized per house in most cases. However, no significant association between house positivity status and a range of factors were identified in univariate analysis (Table S3).

**Table 2.** Risk factor analysis for *S. aureus* colonization amongst household contacts

	Household contact member *S. aureus* status		Univariate		
Variable	Negative, *N*=89*	Positive, *N*=22*	OR	95%** CI**	***P*-value**
Demographics					
Sex					
M	42 (47%)	5 (23%)	–	–	
F	47 (53%)	17 (77%)	3.04	1.09, 9.89	**0.044**
Age					
0–10	13 (15%)	1 (4.5%)	–	–	
10–18	14 (16%)	2 (9.1%)	1.86	0.16, 42.8	0.63
18–35	22 (25%)	5 (23%)	2.95	0.41, 59.9	0.35
35–60	30 (34%)	10 (45%)	4.33	0.71, 83.7	0.18
>60	10 (11%)	4 (18%)	5.20	0.64, 111	0.17
Age (median)	28 (14, 49)	42 (26, 54)	1.02	1.00, 1.05	0.078
Occupation					
Student	25 (28%)	5 (23%)	–	–	
Agriculture	15 (17%)	5 (23%)	1.67	0.40, 6.95	0.47
Unemployed/retired	33 (37%)	11 (50%)	1.67	0.53, 5.85	0.40
Office based	11 (12%)	1 (4.5%)	0.45	0.02, 3.28	0.49
Infant	5 (5.6%)	0 (0%)	0.00		>0.99
Healthcare exposure in the last 12 months					
Hospitalized	3 (3.4%)	0 (0%)	0.00		>0.99
Outpatients	51 (57%)	13 (59%)	1.08	0.42, 2.85	0.88
Medical procedure	5 (5.6%)	0 (0%)	0.00		>0.99
Medical					
Infection at first visit	6 (6.7%)	3 (14%)	2.18	0.43, 9.10	0.30
Diabetes mellitus	1 (1.1%)	1 (4.5%)	4.19	0.16, 109	0.32
Chronic kidney disease	2 (2.2%)	0 (0%)	0.00		>0.99
Skin disease	10 (11%)	4 (18%)	1.76	0.44, 5.93	0.38
Household					
>4 residents	38 (43%)	9 (41%)	0.93	0.35, 2.38	0.88
Proportion positive					
0	61 (69%)	4 (18%)	–	–	
0.25–0.49	26 (29%)	12 (55%)	7.04	2.22, 27.1	**0.002**
0.5–0.74	1 (1.1%)	1 (4.5%)	15.3	0.54, 440	0.070
0.75–0.99	1 (1.1%)	3 (14%)	45.8	4.73, 1,058	**0.003**
1	0 (0%)	2 (9.1%)	238,683,252	0.00, na	>0.99
Animals					
Ownership	86 (97%)	20 (91%)	0.35	0.05, 2.78	0.27
Domestic	83 (93%)	19 (86%)	0.46	0.11, 2.32	0.30
Livestock	14 (16%)	4 (18%)	1.19	0.31, 3.80	0.78
Linked index participant					
Age					
18–35	18 (20%)	8 (36%)	–	–	
35–60	57 (64%)	10 (45%)	0.39	0.13, 1.17	0.089
>60	14 (16%)	4 (18%)	0.64	0.15, 2.49	0.53
Age (median)	45 (39, 56)	47 (22, 52)	0.99	0.96, 1.02	0.63
Sex					
M	64 (72%)	11 (50%)	–	–	
F	25 (28%)	11 (50%)	2.56	0.98, 6.73	0.054
Infection					
BSI	8 (9.0%)	2 (9.1%)	–	–	
Other	10 (11%)	4 (18%)	1.60	0.24, 13.8	0.63
SSTI	71 (80%)	16 (73%)	0.90	0.20, 6.35	0.90
Infection onset					
CA	68 (76%)	17 (77%)	–	–	
HA	21 (24%)	5 (23%)	0.95	0.29, 2.75	0.93
Sequence type					
nt	4 (4.5%)	2 (9.1%)	–	–	
Other	22 (25%)	5 (23%)	0.45	0.07, 3.93	0.43
ST1	6 (6.7%)	2 (9.1%)	0.67	0.06, 7.55	0.73
ST5	13 (15%)	2 (9.1%)	0.31	0.03, 3.23	0.31
ST6	18 (20%)	3 (14%)	0.33	0.04, 3.15	0.30
ST672	21 (24%)	7 (32%)	0.67	0.10, 5.56	0.68
ST772	5 (5.6%)	1 (4.5%)	0.40	0.02, 5.77	0.51
PVL present	21 (24%)	3 (14%)	0.51	0.11, 1.69	0.32

* *n* (%); Median (IQR).

Statistically significant results (*P* < 0.05) are indicated in bold.

BSIBloodstream infectionCACommunity-associatedHAHealthcare-associatedSSTISkin and soft tissue infection

### Characterization of strains

All 101 isolates identified as *S. aureus* by phenotypic methods underwent WGS. This included 77 infecting strains and 24 colonizing strains. Through WGS, 13/101 were subsequently shown to not be *S. aureus* so were excluded from further analysis. These isolates have been previously discussed and included eight IP and five *S. argenteus* isolates. *S. argenteus* is a coagulase-positive staphylococci that is phenotypically indistinguishable from *S. aureus*. All five of these isolates were associated with colonization and were isolated from five unlinked HC. They were all *mecA* and PVL negative, with a sequence type of ST2550. Therefore, a total of 88 isolates were included in the final genomic analysis, including 69 infecting (IP: 42/69 and AP: 27/69) and 19 colonizing (HC: 18/19 and IP: 1/19) strains.

**Fig. 1.**
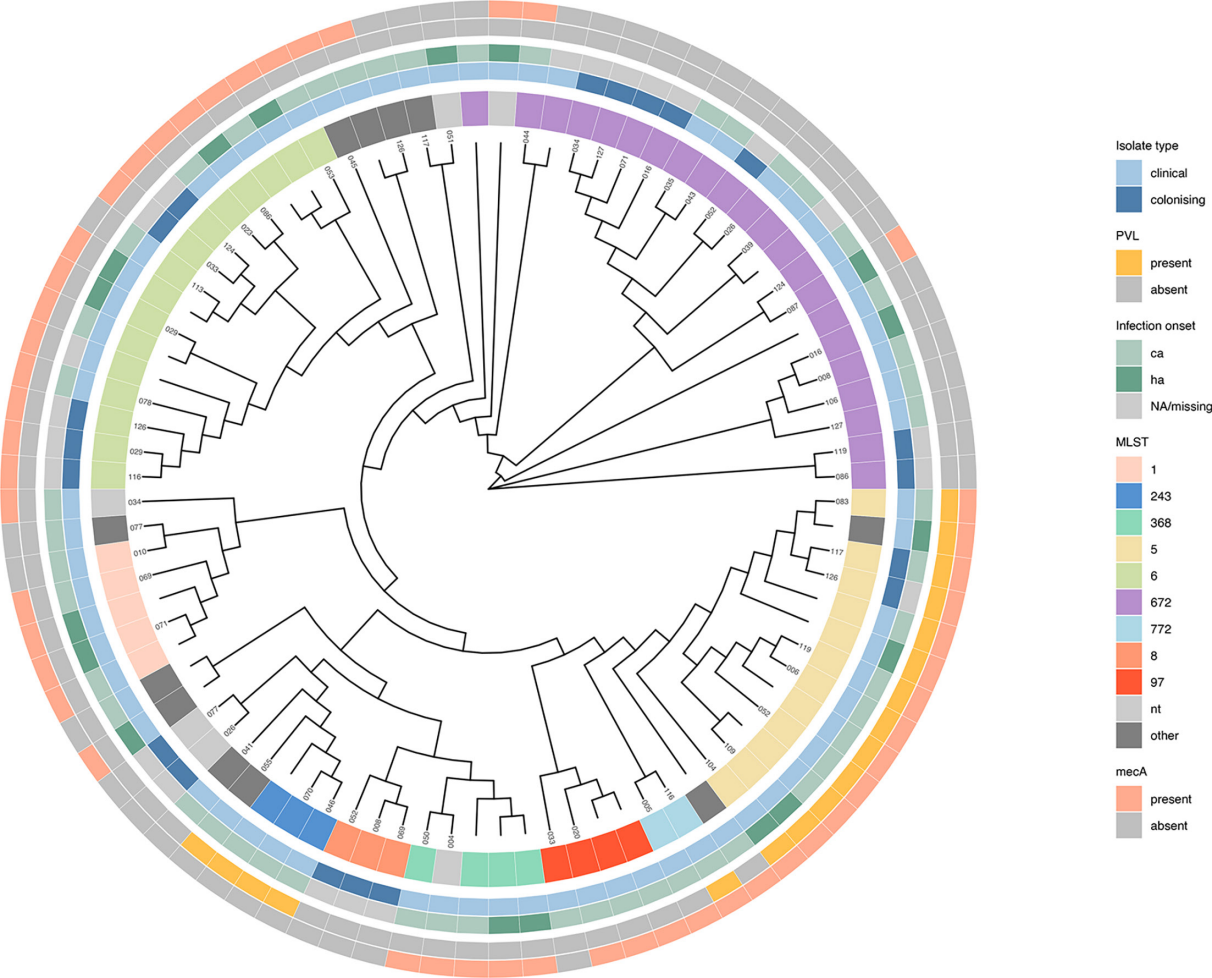
Unrooted maximum likelihood cladogram of 88 *S*. *aureus* genomes and 1672 core genes. The outer rings display clinical and molecular characteristics related to each isolate. The participant’s ‘house identifier number’ is displayed at the tip or left blank for additional participants.

Analysis of the *S. aureus* genomes by MLST was used to identify genetic relationships and group-related isolates by sequence type. Overall, 20 unique MLST were identified with ST672 the most prevalent (22/88, 25.0%) (Figs 1 and S1). Other common sequence types included ST6 (16/88, 18.2%), ST5 (12/88, 13.6%) and ST1 (5/88, 5.7%). The remaining 33 (37.5%) strains were split across 16 other types, and 6.8% (6/88) could not be typed by MLST. No specific sequence type was more commonly associated with infecting or colonizing states. However, there was much greater diversity in the CA infection group with 17 unique sequence types compared with 7 causing HA infections.

Over half of all isolates possessed the *mecA* gene (50/88, 56.8%), which was typically located within the SCC*mec* type IV cassette (40/50, 80.0%). The *mecC* gene was not detected in any isolates. No phenotypic resistance or genotypic markers of vancomycin or mupriocin resistance were detected (including *vanA*, *vanB*, *vanC* and *mupA*). The median mupirocin MIC was onefold higher in clinical isolates compared to colonizing strains (0.13 vs. 0.09 mg l^−1^, *P*=0.012). Overall, PVL genes (both *LukS-PV* and *LukF-PV*) were present in 20.5% (18/88) and nearly universally in infecting isolates (16/18, 88.9%).

### Phylogenetic analysis

Using a cgSNP threshold of 15, WGS analysis did not identify any houses where the IP’s clinical strain was also found colonizing at least one HC. In three houses, the IP and HC strains were genetically similar, with a median 85 SNP differences (IQR 83–89.5), compared with a median 7761 SNP (IQR 7192–8268) in other positive houses.

Whilst there was high genetic diversity in the sequenced strains, three MLST (ST672, ST5 and ST6) predominated and between them accounted for over half of all isolates (50/88, 56.8%). They formed clusters based on common epidemiological, molecular and phenotypic characteristics (Fig. 1). Firstly, whilst all three were identified in both colonized and infected subjects, ST5 strains accounted for a much smaller proportion of colonization cases (Table 3). Secondly, all three caused both CA and HA infection, but ST672 strains were notably more associated with CA infection and ST6 with HA infection (Table 3). However, for all three, the CA- and HA-linked strains were mixed on the cladogram and not clustered by environmental niche (Fig. 1). Finally, the presence of genotypic markers of virulence, including the *mecA* and PVL genes, varied between the three MLST. In particular, ST5 isolates exhibited a more virulent phenotype, with universal presence of PVL (12/12 vs. 0/38, *P*<0.001). The *mecA* gene was detected in nearly all ST5 and ST6 but was notably absent from ST672 (27/28, 96.4% vs. 2/22, 9.1%, *P*<0.001). ST5 isolates typically harboured the SCC*mec* IVc element (11/12, 91.7%), whereas SCC*mec* IVa was more common in ST6 *S. aureus* (14/16, 87.5%).

**Table 3.** Characteristics of ST5, ST6 and ST672 isolates

Characteristics	ST5, *N*=12*	ST6, *N*=16*	ST672, *N*=22*	*P*-value†
Isolate type				0.7
Colonizing	2 (17%)	5 (31%)	7 (32%)	
Infection	10 (83%)	11 (69%)	15 (68%)	
Infection source				0.4
Community-associated	8 (73%)	6 (60%)	11 (85%)	
Healthcare-associated	3 (27%)	4 (40%)	2 (15%)	
Unknown	1	6	9	
*mecA*	12 (100%)	15 (94 %)	2 (9.1 %)	**<0.001**
PVL	12 (100%)	0 (0%)	0 (0%)	**<0.001**
*SCCmec* type				**<0.001**
IIa	0 (0%)	1 (6.3%)	0 (0%)	
IVa	1 (8.3%)	14 (88%)	0 (0%)	
IVc	11 (92%)	0 (0%)	1 (4.5%)	
V	0 (0%)	0 (0%)	1 (4.5%)	
N/A	0 (0%)	1 (6.3%)	20 (91%)	

* *n* (%).

† Fisher’s exact test; Pearson’s chi-squared test.

Statistically significant results (*P* < 0.05) are indicated in bold.

PVLPanton-Valentine leukocidin

### ST672: further characterization

The ST672 type was frequently identified in this cohort and was associated with both infection (15/69, 21.7%) and colonization (7/19, 36.8%). While this strain is increasingly recognized globally, it has not been previously described in Sri Lanka so was characterized further. These isolates were typically staphylococcal protein A (spa) type-3841 (17/22, 77.3%), *mecA* negative (20/22, 90.9%) and PVL negative (22/22, 100%). Eleven of the 15 (73.3%) infecting ST672 isolates were associated with CA infection, similar to all other ST types combined (38/54, 70.4%). Amongst the 15 clinical isolates, no resistance was detected to co-trimoxazole but was detected for ciprofloxacin (12/15, 80%), clindamycin (2/14, 14.3%), erythromycin (8/14, 57.1%) and fusidic acid (2/10, 20.0%). Biofilm formation was quantified in 21 of the ST672 isolates as it is a virulence factor that may play a role in maintaining both infection and colonization [33,34]. They were classified as ‘strong’ in 14/21, ‘moderate’ in 2/21 and ‘weak’ in 5/21. All six colonizing isolates were strong biofilm producers, whereas 7/15 (46.7%) infecting isolates were classified as moderate or weak.

## Discussion

This prospective cohort study has characterized the dynamics and molecular epidemiology of *S. aureus* infection and colonization in Sri Lanka, a LMIC with a high prevalence of MRSA-associated infection. We have demonstrated a broad diversity of strains that are intermixing between community and healthcare settings. Dominant clones were also identified and have been characterized further. By linking longitudinal epidemiological data and a large set of WGS data, this study provides insight into the circulation of *S. aureus* strains between hospital and community environments.

The baseline prevalence of *S. aureus* colonization amongst a healthy Sri Lankan community population (HC) was 11.7% and 3.6% for MRSA. These rates are lower than previous publications from Sri Lanka, with overall colonization reported between 22–42.5% and 4.3–7.4% for MRSA specifically [35,39]. However, none of these studies are directly comparable to our cohort as they include high-risk populations, including farmers, hospital in-patients and students with clinical exposure. However, the prevalence in this study was more in keeping with studies from the wider South Asia region, where *S. aureus* colonization is reported between 6.0 and 29.3% and MRSA between 0 and 4.5% [40,43]. Genomic analysis also revealed five *S. argenteus* isolates that were phenotypically misidentified as *S. aureus*. This is a relatively novel entity, and to the best of our knowledge, this is the first published description of *S. argenteus* in South Asia.

This dataset permits a detailed analysis of *S. aureus* transmission networks in two environmental niches and across both infective and colonized states. Amongst high genetic diversity, we have identified three dominant strains that appear to be circulating freely in both community and healthcare settings. This is further supported by the phylogenetic analysis that identified genetically similar subtypes in both locations, with no distinct clusters. It is, therefore, important to consider the mechanisms and routes by which this mixing occurs as this could inform infection control procedures. We have previously identified a dominant ST5 MRSA clone amongst hospitalized patients, with evidence that it was also circulating more widely in the community [9]. In the USA, the USA300 clone was initially linked with CA infection but is now well established as a healthcare-associated pathogen, and similar transitions have been reported globally [3,44]. Genetic analysis suggests that this may have been driven by a reservoir of community-associated colonization that was subsequently imported into healthcare environments [3,45, 46]. Therefore, it follows that community settings are integral to the maintenance and propagation of these strains. Several studies in North America have documented household transmission of infecting strains, often with secondary episodes of colonization and infection [5,47, 48]. The risk of secondary cases of infection in household contacts of CA *S. aureus* infection can be reduced with topical decolonization therapy [49]. Whilst we were able to demonstrate community circulation of *S. aureus*, colonization with the index’s infecting strain amongst HC was not detected. This may be explained by variability in the dominant clones in these two settings. Equally, differences in cultural and social norms may impact transmission events that require close contact. Similar studies conducted in LMIC are less common and are limited by methodological weaknesses, for example, the use of poorly discriminatory typing methods, limited sample size and/or a cross-sectional design that may miss transient colonization episodes [50,52]. Therefore, the role of the house in maintaining and propagating *S. aureus* circulation in this setting remains unclear. The network of potential sites of community exposure is complex and extends beyond the house. Household decolonization strategies may, therefore, not be as beneficial in this setting.

There is limited data on *S. aureus* epidemiology from South Asia, in particular molecular studies. Where good quality data is available, this region consistently reports some of the highest rates of AMR, including MRSA and vancomycin resistant *S. aureus* [7,8, 53]. We identified a dominant circulating clone that was typically ST672-MSSA-PVL negative and spa-t3841. It was associated with both colonization and infection and appeared to be more successful in community settings. It was not identified in clinical isolates previously collected from the same hospital in 2014 [9]. Instead, the dominant clone at that time was ST5-SCC*mec* IVc-PVL positive and spa-t0002 which was also frequently identified in the current cohort and is increasingly recognized globally [54]. Interestingly, in the current study, this strain seemed less able to establish colonization and was predominantly associated with infection. The underlying cause for this is not clear, although PVL-producing strains may have a predominantly infective as opposed to colonizing phenotype [55]. The emergence of ST672 has been observed over the last decade in neighbouring regions including Asia-Pacific and the Arabian Gulf. Originally reported in Western Australia, and latterly in India and Kuwait, these isolates are variably methicillin resistant (with SCC*mec* IV or V) and are split across three spa types (t1309, t3840 and t3841). They are typically PVL negative and possess agr type I and capsular polysaccharide 8 [56,59]. Similar characteristics were observed in our cohort of ST672 strains, although the most striking difference was the near-universal absence of the *mecA* gene. Although MSSA ST672 strains have previously been documented in South Asia, they are less common than their methicillin-resistant counterparts [57,58]. This distinction in *mecA* status may suggest a non-linked lineage of ST672 in this cohort.

This study was designed primarily as an exploratory epidemiological investigation to assess *S. aureus* colonization and infection in a region where such data are scarce. We aimed to provide a representative cross-section of the population and capture the genomic diversity of *S. aureus.* However, several limitations to this study should be acknowledged. Firstly, the average number of household members was lower than anticipated, which may have limited recruitment and our ability to detect household transmission events. Furthermore, the fieldwork was conducted during great social disruption due to the COVID-19 pandemic. We suspect that shifts in social behaviour around this time (e.g. isolation, face mask wearing and increased hand hygiene) would have had an unquantifiable impact on *S. aureus* transmission. The pandemic also resulted in unforeseen delays between IP recruitment and initial HC sampling as well as between V1 and V2, potentially leading to missed episodes of colonization. Secondly, sampling at two timepoints over a large time interval will have missed transient episodes of colonization, particularly of infecting strains. In one cohort, the average duration of MRSA colonization was only 21 days following successful treatment for MRSA SSTI [60]. This, however, was a pragmatic decision due to limited resources. Future work would be enhanced by a longer period of follow-up with more frequent sampling timepoints. Thirdly, we are not able to comment on the direction of transmission, and our methodology assumes that the infecting strain was first encountered by the IP rather than a HC. Finally, we did not sample animals or the environment, and both have been shown to play a role in both introduction and transmission events within the household [5]. This consideration is particularly relevant for this population, which has a high proportion of animal ownership.

## Conclusion

*S. aureus* is now well established globally as a community- and healthcare-associated pathogen. Some strains are successful at forming reservoirs in both environments, and this serves to maintain a cycle of transmission. A detailed understanding of localized *S. aureus* epidemiology and transmission networks is, therefore, vital to inform intervention strategies. This is particularly important for many LMIC where high rates of AMR are combined with limited resources. In this study, we documented the success of a small number of genetically related strains in both infection and colonization, without distinction between community and healthcare settings. However, we did not find evidence of the household acting as a specific focus for transmission of infecting strains. Accordingly, expensive decolonization strategies that target the household may be of less value in this setting. Instead, further work is now required to delineate these community transmission networks and risk factors in greater detail.

## supplementary material

10.1099/mgen.0.001336 Supplementary Material 1.

## Abbreviations

AMR: antimicrobial resistance
AP: additional participants
CA: community-associated
CI: confidence interval
HA: healthcare-associated
HC: household contacts
IP: index participants
IQR: interquartile range
LMIC: low- and middle-income countries
MIC: minimum inhibitory concentration
MLST: multilocus sequence typing
MRSA: methicillin-resistant *S. aureus*
MSA: mannitol salt agar
OR: odds ratio
PVL: Panton–Valentine leukocidin
SSTI: skin and soft tissue infection
ST: sequence type
TSB: tryptic soy broth
WGS: whole-genome sequencing
