# Supplementary material for: A prospective cohort study to investigate the transmission and burden of Staphylococcus aureus in Sri Lanka
Source: Microb Genom. 2024 Dec 19;10(12):001336. doi: 10.1099/mgen.0.001336 (PMC11657565; doi:10.1099/mgen.0.001336)
Supplement: Supplementary Material 1. [file mgen-10-01336-s001.pdf]

Figure S1: Unrooted phylogenetic tree of 88 S. aureus genomes and 1672 core genes

The tips are labelled with the MLST type. The participant´s ‘house identifier number´ is displayed in a column at the far right or left blank for Additional Participants

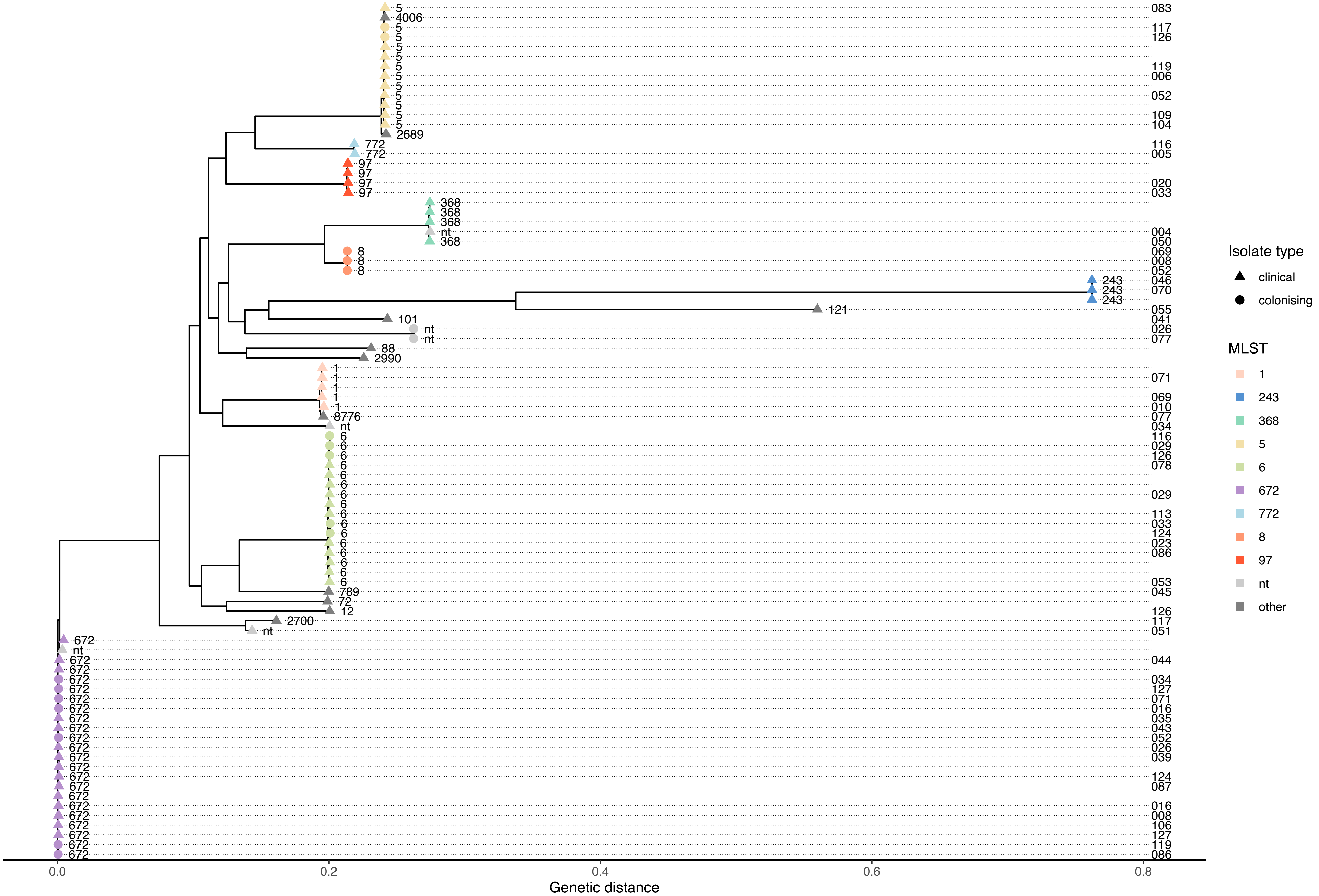

Participant ID	Isolate	NCBI BioSample number	Participant type	Age	Sex	Isolate type	Infection type	Infection onset	MLST	spa type	*mecA* present	*SCCmec* type	PVL present	TSST present
Staphylococcus aureus														
0099	057364_SL099	SAMN40655903	Additional	48	Male	Clinical	Skin and soft tissue	Community	2689	nt	y	IVa	n	n
0027	057424_SL027	SAMN40655941	Additional	35	Male	Clinical	Skin and soft tissue	Healthcare	6	t304	y	IVa	n	n
0001	057430_SL001	SAMN40655944	Additional	57	Male	Clinical	Isolated bacteraemia	missing	6	t6618	y	IVa	n	n
0002	057431_SL002	SAMN40655945	Additional	23	Female	Clinical	Respiratory tract	Community	2990	t091	n	NA	n	n
0009	057432_SL009	SAMN40655946	Additional	52	Male	Clinical	Isolated bacteraemia	Healthcare	4006	t002	y	IVc	y	n
0012	057433_SL012	SAMN40655947	Additional	36	Male	Clinical	Surgical site	Community	97	t4173	y	IVc	n	n
0014	057437_SL014	SAMN40655950	Additional	21	Female	Clinical	Skin and soft tissue	Community	6	t304	y	IVa	n	n
0022	057438_SL022	SAMN40655951	Additional	missing	Male	Clinical	Skin and soft tissue	Healthcare	368	t425	y	IIIa	n	n
0048	057447_SL048	SAMN40655958	Additional	missing	Female	Clinical	Skin and soft tissue	missing	672	t1240	n	NA	n	n
0062	057448_SL062	SAMN40655959	Additional	54	Male	Clinical	Isolated bacteraemia	Community	368	t425	y	IIIa	n	n
0074	057449_SL074	SAMN40655960	Additional	56	Male	Clinical	Skin and soft tissue	Healthcare	nt	t315	y	IVh	n	n
0084	057450_SL084	SAMN40655961	Additional	52	Male	Clinical	Isolated bacteraemia	Healthcare	88	t186	y	IVa	n	n
0092	057451_SL092	SAMN40655962	Additional	69	Female	Clinical	Skin and soft tissue	Community	672	t3841	n	NA	n	n
0094	057452_SL094	SAMN40655963	Additional	36	Male	Clinical	Skin and soft tissue	Community	72	t4897	y	IVc	n	y
0098	057453_SL098	SAMN40655964	Additional	48	Female	Clinical	Skin and soft tissue	Community	5	t1062	y	IVc	y	n
0103	057454_SL103	SAMN40655965	Additional	58	Male	Clinical	Skin and soft tissue	Healthcare	368	t425	y	IIIa	n	n
0115	057455_SL115	SAMN40655966	Additional	35	Male	Clinical	Surgical site	Healthcare	6	t304	y	IVa	n	n
0118	057456_SL118	SAMN40655967	Additional	39	Male	Clinical	Surgical site	Healthcare	1	t127	y	IVa	n	n
0128	057457_SL128	SAMN40655968	Additional	35	Female	Clinical	Isolated bacteraemia	Community	1	t127	y	IVa	n	n
0040	057463_SL040	SAMN40655971	Additional	37	Female	Clinical	Ear, nose, throat	missing	672	t3841	n	NA	n	n
0067	057464_SL067	SAMN40655972	Additional	48	Female	Clinical	Isolated bacteraemia	Community	97	t267	y	IVc	n	n
0075	057465_SL075	SAMN40655973	Additional	25	Male	Clinical	Skin and soft tissue	Community	5	t002	y	IVc	y	n
0080	057466_SL080	SAMN40655974	Additional	26	Male	Clinical	Skin and soft tissue	Community	243	t021	n	NA	y	n
0085	057467_SL085	SAMN40655975	Additional	71	Male	Clinical	Isolated bacteraemia	Healthcare	5	t002	y	IVc	y	n
0088	057468_SL088	SAMN40655976	Additional	36	Male	Clinical	Surgical site	Community	672	t003	n	NA	n	n
0095	057470_SL095	SAMN40655977	Additional	59	Female	Clinical	Isolated bacteraemia	Community	6	t304	y	IVa	n	n
0112	057471_SL112	SAMN40655978	Additional	34	Female	Clinical	Gynaecological	Community	5	t002	y	IVc	y	n
16C1	057399_SL144	SAMN40655928	Household contact	46	Female	Colonising	NA	NA	672	t3841	n	NA	n	n
86C3	057401_SL149	SAMN40655929	Household contact	63	Male	Colonising	NA	NA	672	t3841	n	NA	n	n
77C3	057403_SL152	SAMN40655930	Household contact	20	Female	Colonising	NA	NA	nt	t18585	n	NA	n	n
127C1	057405_SL155	SAMN40655931	Household contact	66	Female	Colonising	NA	NA	672	t3841	n	NA	n	n

Participant ID	Isolate	NCBI BioSample number	Participant type	Age	Sex	Isolate type	Infection type	Infection onset	MLST	spa type	*mecA* present	*SCCmec* type	PVL present	TSST present
124C3	057407_SL157	SAMN40655932	Household contact	17	Female	Colonising	NA	NA	6	t304	y	IIa	n	n
126C4	057412_SL164	SAMN40655934	Household contact	9	Female	Colonising	NA	NA	5	t2051	y	IVc	y	n
8C2	057413_SL165	SAMN40655935	Household contact	58	Female	Colonising	NA	NA	8	t008	n	NA	n	n
71C1	057414_SL166	SAMN40655936	Household contact	46	Female	Colonising	NA	NA	672	t3841	n	NA	n	n
33C1	057416_SL168	SAMN40655937	Household contact	61	Female	Colonising	NA	NA	6	t304	y	IVa	n	n
119C1	057417_SL171	SAMN40655938	Household contact	56	Male	Colonising	NA	NA	672	t3841	n	NA	n	n
69C3	057420_SL176	SAMN40655939	Household contact	25	Male	Colonising	NA	NA	8	t008	n	NA	n	n
34C1	057421_SL177	SAMN40655940	Household contact	43	Female	Colonising	NA	NA	672	t3841	n	NA	n	n
52C2	057425_SL154	SAMN40655942	Household contact	38	Female	Colonising	NA	NA	8	t008	n	NA	n	n
126C3	057442_SL159	SAMN40655954	Household contact	15	Female	Colonising	NA	NA	6	t2196	y	IVa	n	n
29C1	057444_SL170	SAMN40655955	Household contact	28	Female	Colonising	NA	NA	6	t2196	y	IVa	n	n
52C2	057445_SL174	SAMN40655956	Household contact	38	Female	Colonising	NA	NA	672	t3841	n	NA	n	n
116C1	057458_SL181	SAMN40655969	Household contact	39	Female	Colonising	NA	NA	6	t2196	y	IVa	n	n
26C1	057460_SL185	SAMN40655970	Household contact	63	Female	Colonising	NA	NA	nt	t18585	n	NA	n	n
0005	057352_SL005	SAMN40655892	Index	43	Male	Clinical	Skin and soft tissue	Community	772	t657	y	UT	n	n
0006	057353_SL006	SAMN40655893	Index	62	Male	Clinical	Skin and soft tissue	Community	5	t002	y	IVc	y	n
0008	057354_SL008	SAMN40655894	Index	79	Female	Clinical	Skin and soft tissue	Community	672	t3175	n	NA	n	n
0020	057356_SL020	SAMN40655895	Index	42	Male	Clinical	Skin and soft tissue	Community	97	t521	y	IVa	n	n
0026	057357_SL026	SAMN40655896	Index	33	Male	Clinical	Skin and soft tissue	Community	672	t3841	n	NA	n	n
0041	057358_SL041	SAMN40655897	Index	48	Female	Clinical	Skin and soft tissue	Community	101	nt	n	NA	n	n
0050	057359_SL050	SAMN40655898	Index	29	Male	Clinical	Skin and soft tissue	Community	368	t425	y	IIIa	n	n
0051	057360_SL051	SAMN40655899	Index	49	Male	Clinical	Surgical site	Healthcare	nt	nt	n	NA	n	n
0053	057361_SL053	SAMN40655900	Index	39	Male	Clinical	Skin and soft tissue	Community	6	t304	y	IVa	n	n
0070	057362_SL070	SAMN40655901	Index	58	Male	Clinical	Skin and soft tissue	Community	243	t021	n	NA	y	n
0086	057363_SL086	SAMN40655902	Index	44	Female	Clinical	Respiratory tract	Healthcare	6	t304	y	IVa	n	n
0109	057365_SL109	SAMN40655904	Index	65	Male	Clinical	Skin and soft tissue	Healthcare	5	t002	y	IVc	y	n
0113	057367_SL113	SAMN40655905	Index	41	Female	Clinical	Skin and soft tissue	Community	6	t304	n	NA	n	n
0069	057374_SL140	SAMN40655906	Index	20	Female	Clinical	Skin and soft tissue	Community	1	t127	y	IVa	n	n
0004	057378_SL004	SAMN40655909	Index	53	Female	Clinical	Skin and soft tissue	Community	nt	t425	y	IIIa	n	n
0029	057380_SL029	SAMN40655910	Index	65	Female	Clinical	Skin and soft tissue	Healthcare	6	t304	y	IVa	n	n
0033	057381_SL033	SAMN40655911	Index	25	Female	Clinical	Gynaecological	Community	97	t267	n	NA	n	y
0034	057382_SL034	SAMN40655912	Index	21	Male	Clinical	Skin and soft tissue	Community	nt	t3841	y	V	n	n

Participant ID	Isolate	NCBI BioSample number	Participant type	Age	Sex	Isolate type	Infection type	Infection onset	MLST	spa type	*mecA* present	*SCCmec* type	PVL present	TSST present
0046	057383_SL046	SAMN40655913	Index	45	Male	Clinical	Skin and soft tissue	Community	243	t021	n	NA	y	n
0052	057384_SL052	SAMN40655914	Index	67	Male	Clinical	Skin and soft tissue	Community	5	t002	y	IVc	y	n
0055	057385_SL055	SAMN40655915	Index	38	Male	Clinical	Skin and soft tissue	Community	121	t3184	n	NA	y	n
0071	057386_SL071	SAMN40655916	Index	52	Male	Clinical	Surgical site	Healthcare	1	t127	y	IVa	n	n
0077	057387_SL077	SAMN40655917	Index	52	Male	Clinical	Skin and soft tissue	Community	8776	t127	n	NA	n	n
0083	057388_SL083	SAMN40655918	Index	44	Male	Clinical	Skin and soft tissue	Community	5	t002	y	IVc	y	n
0087	057389_SL087	SAMN40655919	Index	30	Male	Clinical	Surgical site	Healthcare	672	t3841	y	V	n	n
0106	057390_SL106	SAMN40655920	Index	55	Female	Clinical	Skin and soft tissue	Community	672	t3841	n	NA	n	n
0116	057391_SL116	SAMN40655921	Index	50	Male	Clinical	Skin and soft tissue	Community	772	t657	y	UT	y	n
0117	057392_SL117	SAMN40655922	Index	56	Male	Clinical	Skin and soft tissue	Community	2700	t1931	n	NA	n	n
0119	057393_SL119	SAMN40655923	Index	44	Female	Clinical	Skin and soft tissue	Community	5	t002	y	IVc	y	n
0124	057394_SL124	SAMN40655924	Index	51	Female	Clinical	Skin and soft tissue	Community	672	t3841	n	NA	n	n
0126	057395_SL126	SAMN40655925	Index	20	Female	Clinical	Skin and soft tissue	Community	12	t14589	n	NA	n	n
0127	057396_SL127	SAMN40655926	Index	20	Male	Clinical	Skin and soft tissue	Community	672	t3841	n	NA	n	n
0078	057398_SL141	SAMN40655927	Index	19	Male	Clinical	Skin and soft tissue	Community	6	t304	y	IVa	n	n
0043	057434_SL043	SAMN40655948	Index	59	Male	Clinical	Isolated bacteraemia	Community	672	t3841	n	NA	n	n
0023	057439_SL023	SAMN40655952	Index	45	Male	Clinical	Skin and soft tissue	Community	6	t304	y	IVa	n	n
0104	057441_SL104	SAMN40655953	Index	58	Male	Clinical	Isolated bacteraemia	Healthcare	5	t002	y	IVa	y	n
0045	057446_SL045	SAMN40655957	Index	62	Male	Clinical	Skin and soft tissue	Community	789	t8655	y	IVa	n	n
0010	057472_SL010	SAMN40655979	Index	18	Male	Clinical	Skin and soft tissue	Community	1	t948	n	NA	n	n
0035	057473_SL035	SAMN40655980	Index	65	Male	Clinical	Skin and soft tissue	Community	672	t3841	n	NA	n	n
0039	057474_SL039	SAMN40655981	Index	72	Female	Clinical	Skin and soft tissue	Community	672	t3841	n	NA	n	n
0044	057475_SL044	SAMN40655982	Index	41	Female	Clinical	Skin and soft tissue	Community	672	t1240	y	IVc	n	n
0016	057479_SL132	SAMN40655983	Index	48	Male	Clinical	Isolated bacteraemia	Healthcare	672	t3175	n	NA	n	n
0117	057482_SL175	SAMN40655984	Index	56	Male	Colonising	NA	Community	5	t2051	y	IVc	y	n
Staphylococcus argenteus														
51C2	057376_SL147	SAMN40655907	Household contact	18	Male	Colonising	NA	NA	2250	t6675	n	NA	n	n
43C3	057377_SL150	SAMN40655908	Household contact	33	Female	Colonising	NA	NA	2250	t6675	n	NA	n	n
126C2	057408_SL158	SAMN40655933	Household contact	48	Female	Colonising	NA	NA	2250	t6675	n	NA	n	n
8C3	057426_SL169	SAMN40655943	Household contact	47	Male	Colonising	NA	NA	2250	t6675	n	NA	n	n
23C1	057436_SL180	SAMN40655949	Household contact	41	Female	Colonising	NA	NA	2250	t6675	n	NA	n	n

TABLE S1 Additional information on all sequenced *S. aureus* isolates

**Infection site**	**Community acquired**, N = 34[1]	**Hospital acquired**, N = 8[1]
blood stream	1 (2.9%)	2 (25%)
gynaecological	1 (2.9%)	0 (0%)
respiratory tract	0 (0%)	1 (13%)
skin and soft tissue	32 (94%)	2 (25%)
surgical site	0 (0%)	3 (38%)

[1]n (%)

TABLE S2 Anatomical site of infection by onset of infection for index participants

Variable	Household *S.aureus* status		Univariate		
	**neg**, N = 23[1]	**pos**, N = 19[1]	**OR**[2]	**95% CI**[2]	**p-value**
**Household**					
>4 residents	5 (22%)	7 (37%)	2.10	0.54, 8.63	0.29
>2 adults	11 (48%)	13 (68%)	2.36	0.68, 8.82	0.18
>=1 child	13 (57%)	10 (53%)	0.85	0.25, 2.92	0.80
house material quality					
low	15 (65%)	12 (63%)	—	—	
medium	4 (17%)	5 (26%)	1.56	0.34, 7.58	0.56
high	4 (17%)	2 (11%)	0.63	0.08, 3.79	0.62
water supply					
piped	5 (22%)	4 (21%)	—	—	
filtered	17 (74%)	13 (68%)	0.96	0.21, 4.54	0.95
natural	1 (4.3%)	2 (11%)	2.50	0.17, 66.6	0.51
**Animals**					
ownership	22 (96%)	17 (89%)	0.39	0.02, 4.36	0.45
single	12 (52%)	13 (68%)	1.99	0.57, 7.38	0.29
multiple	11 (48%)	6 (32%)	0.50	0.14, 1.75	0.29
domestic	22 (96%)	16 (84%)	0.24	0.01, 2.09	0.24
livestock	3 (13%)	4 (21%)	1.78	0.34, 10.2	0.49
**Household member medical issue in last 12 months**					
hospitalised	15 (65%)	9 (47%)	0.48	0.13, 1.65	0.25
outpatients	23 (100%)	18 (95%)	0.00		>0.99
infection	7 (30%)	7 (37%)	1.33	0.36, 4.93	0.66

[1]n (%)

[2]OR = Odds Ratio, CI = Confidence Interval

TABLE S3 Risk factor analysis for household positivity (at least one member colonised with *S. aureus*)
